# Association of dietary habits and parental-reported sleep tooth grinding with tooth wear in children with mixed dentition

**DOI:** 10.1186/s12903-017-0447-5

**Published:** 2017-12-20

**Authors:** Claudia Restrepo, Daniele Manfredini, Ruben Manrique, Frank Lobbezoo

**Affiliations:** 1 CES-LPH Research Group, Universidad CES, Calle 10 A No. 22-04, Medellín, Colombia; 2 Oral Physiology, School of Dentistry University of Siena, Siena, Italy; 3 Investigations and Innovation Department, Universidad CES, Calle 10 A No. 22-04, Medellín, Colombia; 4 Department of Oral Kinesiology, Academic Centre for Dentistry Amsterdam (ACTA), University of Amsterdam and Vrije Universiteit Amsterdam, Gustav Mahlerlaan 3004, 1081 LA Amsterdam, The Netherlands

**Keywords:** Tooth wear, Sleep Bruxism, Dietary habits, Children

## Abstract

**Background:**

Tooth wear has a multifactorial etiology, thus it should be assessed within a multiple-variable framework. The objective of this investigation was to assess the association of dietary habits and parental-reported sleep tooth grinding (STG) with tooth wear in children with mixed dentition.

**Methods:**

One hundred twenty-one (*N* = 121) subjects (mean age 9.6 years) participated in a cross-sectional study. Wear of 1637 teeth was evaluated using the screening module of the Tooth Wear Evaluation System (TWES). Parental-report of STG was evaluated by means of the Children’s Sleep Habits Questionnaire (CSHQ), whilst dietary habits were investigated by means of the Health Behaviour in School-Aged Children Food-Frequency Questionnaire (HBSC-FFQ). Data were analyzed with the Spearman correlation test and ordinal-multiple-variable regression analyses. Odds Ratio (OR) and ordinal OR were obtained for the independent variables included in the models.

**Results:**

Parental-report of STG is not associated with tooth wear in the mixed dentition; some dietary habits were found to be correlated with specific tooth wear patterns, but the correlation values were weak. Associations were found between dietary habits and the increase-to-increase severity of occlusal/incisal and non-occlusal/non-incisal tooth wear of some teeth (OR > 2).

**Conclusions:**

A strong correlation of dietary habits and sleep tooth grinding with tooth wear in the mixed dentition was not demonstrated. However, dietary habits showed to have effects in terms of increase-to-increase severity.

## Background

Tooth wear is defined as “loss of the tooth substance by chemical or mechanical processes” (http://www-ncbi-nlm-nih.gov/mesh/?term=tooth+wear). During childhood, tooth wear increases with age [1], until reaching a prevalence of 80% by the end of the primary dentition [2] whilst decreasing to about 30% in adolescents with permanent dentition [1].

Wear of the teeth is the result of combined progression of erosion, attrition, and abrasion [3]. Erosion has been associated with intrinsic or extrinsic acids. For instance, subjects consuming large quantities of sweets, non-natural juices, citric fruits, and carbonated drinks are more at risk of developing tooth erosion [4]. On the other hand, attrition is likely more prevalent in subjects with grinding-type bruxism habits, whilst abrasion requires the wearing action of an external object (e.g., a tooth brush) [3]. However, the different types of tooth wear are rarely present alone. Furthermore, when an acid environment is present, there is an increased risk of having all types of tooth wear [3].

Due to its multifactorial etiology, tooth wear should be assessed within a multiple-variable framework [3], including an evaluation of possible provoking conditions, such as dietary habits, soft-drink intake [5], and oral habits like tooth grinding [6, 7].

The literature is plenty of reports on the relationship between dietary habits and tooth wear in children and adolescents [8–11]. Some other studies have also assessed tooth wear in children with sleep tooth grinding (STG) [6, 10]. However, despite such an amount of works, there are only few investigations based on a multifactorial evaluation of tooth wear. Based on the above premises, the aim of this investigation therefore was to assess the association of dietary habits and parental-report of STG with tooth wear in children with mixed dentition.

## Methods

### Study sample

The sample size was calculated based on the data obtained by Huynh et al., for the prevalence of tooth wear in non-bruxist and bruxist children, which was 28.4% and 35.8% respectively [11]. To reach a power of 90% and confidence of 95%, around 800 teeth were needed in each group. Thus, the study was designed to plan evaluation of 1600 teeth.

Based on the above, 133 children aged 6-to-12 years (63 girls and 70 boys) were originally recruited from among the attendants of the clinic of the postgraduate program of Pediatric Dentistry at Universidad CES in Medellín, Colombia. The inclusion criteria were absence of neurologic or psychiatric disorders, reflux disease (GERD), absence of cavitated caries lesions, absence of any previous or current treatment with orthodontics or orofacial orthopedics, presence of normal facial morphology (e.g., absence of cleft lip and palate or other syndromes grossly affecting facial morphology), skeletal class I, according to Colombian standards [12], absence of occlusal open or deep anterior bite, and anterior and posterior crossbites.

The study design provided that all subjects were assessed as far as the below outcome (tooth wear) and associated variables (parental-report STG and dietary habits) are concerned.

### Tooth wear assessment

Tooth wear was evaluated after drying the teeth, while the children were sitting on a dental chair under the same conditions of light.

Tooth wear was scored clinically on a tooth-by-tooth basis with the use of three instruments [13, 14], described in Table 1.

**Table 1:** Prevalence of occlusal/incisal tooth wear and non-occlusal/non-incisal tooth wear in permanent and deciduous teeth

Occlusal/Incisal Tooth Wear	
Grading Scale	Number of teeth with tooth wear (%)
Permanent teeth (*n* = 637)^a^	
0	575 (90.3)
1a	59 (9.3)
1b	1 (0.1)
1c	0 (0)
2	2 (0.3)
3a	0 (0)
3b	0 (0)
4	0 (0)
Deciduous teeth (*n* = 291)^b^	
0	126 (43.3)
1	132 (45.4)
2	27 (9.3)
3	6 (2.1)
4	0 (0)
Non-occlusal/Non-Incisal tooth wear	
Grading scale^25^	Number of teeth with tooth wear (%)
Permanent teeth (*n* = 430)^c^	
0	400 (93.0)
1	29 (6.7)
2	1 (0.2)
Deciduous teeth (*n* = 279)^c^	
0	234 (83.8)
1	34 (12.1)
2	11 (3.9)

^a^Occlusal/Incisal tooth wear in permanent teeth: 0 = no wear; 1a = minimal wear within the enamel of cusps or incisal tips; 1b = Facets within the enamel parallel to the normal planes or contour; 1c = Noticeable flattening of cusps or incisal edges within the enamel; 2 = Wear with dentine exposure and loss of clinical crown high <1/3; 3a = Wear with dentine exposure and loss of clinical crown high of 1/3 – ½; 3b = Wear with dentine exposure and loss of clinical crown of ½- 2/3 and 4 = Wear with dentine exposure and loss of clinical crown of >2/3

^b^Occlusal/Incisal tooth wear in deciduous teeth: 0 = no wear; 1 = visible wear within the enamel; 2 = visible wear with dentin exposure and loss of clinical crown height of ≤1⁄3; 3 = loss of crown height > 1⁄3 but <2⁄3; and 4 = loss of crown height ≥ 2⁄3

^c^Non-Occlusal/Non-Incisal tooth wear in permanent and deciduous teeth: 0 = No wear; 1 = Wear confined to the enamel; 2 = Wear into the dentine

The clinical tooth wear assessment was performed by six undergraduate fourth-year dental students, previously calibrated in preliminary evaluation sessions performed before the beginning of the actual study. Before the onset of the study, the students underwent a three-session instruction, training, and calibration procedure, performed by one of the study authors (C.R.). For that purpose, two steps were followed. First, the leading investigator (C.R) explained and showed the assessment procedure on four trial subjects having the same characteristics of the study participants, but not taking part to the successive calibration step. Second, after the instruction session, two calibration sessions were performed on eighteen children (14 boys and 4 girls; 6-10 years old; mean age 9.1 ± 3 years). For calibration purposes, children were selected based on the presence of all tooth wear categories, included in the classifications by Lobbezoo and Naeije (2001) [13] and the Tooth Wear Evaluation System (TWES) [14].

Each of the 18 children taking part to the calibration procedure were assessed twice by the seven observers (i.e., the six dental students and the leading investigator), 1 week apart. The subjects who were examined during the training and calibration procedures were not included in the final study sample.

The overall inter-rater and intra-rater reliability values (Cohen’s kappa) for clinical occlusal/incisal tooth wear scores ranged between k = 0.81 and k = 1.00. For the non-occlusal/non-incisal/ tooth wear, the overall k-values ranged between 0.94 and 1.00. The tooth with the lowest inter-rater score in both sessions was the first permanent molar for the occlusal tooth wear, and the deciduous canine for the non-occlusal /non-incisal tooth wear. The intra-rater scores, both for primary and permanent dentitions, for occlusal/incisal and non-occlusal/non-incisal tooth wear and for all tooth types, were all above 0.95.

After the calibration process, each child was evaluated by a single undergraduate student. The examination always started with the inspection of the last present molar in the second dental quadrant (left maxillary dental arch) and ended with the last present molar in the third dental quadrant (left mandibular dental arch). The FDI World Dental Federation notation was used to designate the permanent and deciduous teeth [15].

### Report of STG in the children’s sleep habits questionnaire (CSHQ)

The CSHQ is a 35-item, parent-rated questionnaire that assesses the frequency of sleep habits. The evaluation of parental-report of STG in this study was based on an item of the spanish version of the CSHQ, answered by parents (i.e., “How frequently does your child grind/gnash the teeth during sleep?”) [16]. The question has three possible answers, based on an ordinal Likert-type scale that refers to the frequency of nights with parental-reported STG during the week preceding the questionnaire report: Rarely (0-1 night), Sometimes (2–4 nights), and Usually (5–7 nights) [17].

### Dietary habits

To evaluate the dietary habits, the Health Behaviour in School-Aged Children Food-Frequency Questionnaire (HBSC-FFQ) was used. The HBSC-FFQ is a 15-item module included within the HBSC questionnaire. The validation values can be found for each item in the original publication [18].

The question, “How many times a week do you usually eat/drink…?” was followed by the following list of food and beverage items: important sources of fibers (fruits and vegetables), cereals (corn flakes, choco pops, muesli, etc.), breads (white and brown Bread), important sources of calcium (semi-skimmed milk, whole-fat milk, cheese, other milk products (e.g. yoghurt, quark, chocolate milk, fristi, puddings, etc.), and items relevant to youth food culture (crisps, chips, sweets and chocolates, soft drinks, diet soft drinks, alcoholic beverages). The answers were given by the parents in a Likert-type scale (never, less than once a week, once a week, 2-4 days a week, 5-6 times a week, once a day every day, more than once every day).

### Statistical analysis

A selection of teeth for the analysis of wear was performed. Selection was based on the choice of not including adjacent or occluding teeth and including both permanent and deciduous teeth in order to minimize the dependency of the scores on adjacency of teeth [19].

For occlusal/incisal evaluation, teeth 16, 36, 21, 12, 41, 32, 64, 53, 73 and 75 were included, whilst for non-occlusal/non-incisal evaluation, teeth 26, 46, 11, 31, 54, 63, 85 and 83 were selected.

Also for statistical purposes, foods were grouped according to the Food Guide Pyramid in fruits and vegetables, breads and cereals, milk products, fried products, sweets and chocolates, and sodas and alcohol (Food Guide Pyramid. 2010. https://www.cnpp.usda.gov/sites/default/files/archived_projects/FGPPamphlet.pdf).

All main variables, viz., tooth wear, parental-reported STG, and dietary habits, were managed as ordinal variables. The correlations of tooth wear with age, gender, parental-reported STG, and each dietary habit were assessed by using Spearman correlation analysis (Rho).

Ordinal multiple-variable logistic regression analysis was then performed to identify models that best predicted the presence and severity of occlusal/incisal and non-occlusal/non-incisal tooth wear of each of the selected teeth (dependent variables).

To avoid including non-relevant independent variables in the initial model (i.e., age, gender, parental-reported STG, dietary habits), only those variables that were significant at *p* < 0.1 in the single-variable analysis (i.e., Spearman correlation test), were entered in the first step of the ordinal multiple-variable analyses. A backward stepwise procedure was adopted to remove step-by-step the variable with the weakest association, until all variables that were retained in each model had a *p* value <0.05. The odds ratio (OR) and ordinal odds ratios (ordinal OR) for occlusal/incisal and non-occlusal/non-incisal wear of each selected tooth were assessed for each significant factor. According to current interpretations, they were considered clinically relevant if OR ≥ 2 and *p* < 0.05 [20].

The Pseudo r-square (R^2^) was obtained as an estimation of the total variance for occlusal/incisal and non-occlusal/non-incisal tooth wear of each tooth, explained by the significant variables in the models.

## Results

Twelve (*n* = 12) of the 133 healthy children initially recruited for the study were excluded due to the presence of cavitated tooth caries, so that one hundred twenty-one subjects (53 girls and 68 boys; mean age 9.6 years, range 6-12) actually took part to the study. A total of 2780 teeth were assessed, those from which 1637 teeth were considered for data analysis (Table 1).

### Severity of tooth wear in permanent and deciduous teeth

Despite the fact that the majority of evaluated teeth (permanent and deciduous) did not present any wear (*n* = 1335, 81.5%), different severities of tooth wear were observed for both permanent and deciduous teeth. However, in the permanent teeth, there were only a few teeth included in each category of occlusal/incisal wear (Table 1). Due to this reason, the occlusal/incisal wear in the permanent dentition was regrouped into the codes 0 = No wear; 1 = Wear within the enamel; 2 = Wear with dentine exposure, in order to have a more representative sample for statistical analysis. The original severity of wear in the evaluated teeth is presented in Table 1.

### Correlation between age and gender and tooth wear

Positive statistically significant correlations (*p* < 0.05) were found for age and occlusal/incisal wear of 64 and 53 (Table 2); and for age and non-occlusal/non-incisal wear of 26, 46, 54, 85, 63 and 83 (Table 3). However, correlation coefficients were low, with Rho values ranging between 0.20 and 0.42. As for gender, no correlations were found with tooth wear.

**Table 2:** Correlation of age, gender, sleep tooth grinding and dietary-habits with occlusal/incisal tooth wear in permanent and deciduous teeth. Spearman correlation analysis (Rho Correlation Coefficients and *p*-values)

	Occlusal/Incisal tooth wear																			
Variables	16		36		21		12		41		32		64		53		73		75	
	Rho	*p* value	Rho	*P* value	Rho	*p* value	Rho	*p* value	Rho	*p* value	Rho	*p* value	Rho	*p* value	Rho	*p* value	Rho	*p* value	Rho	*p* value
Age	−0.03	0.77	0.2	0.10	0.01	0.86	−0.06	0.57	0.5	0.12	0.02	0.80	0.2	0.04	0.3	0.005	0.1	0.28	0.1	0.34
Gender	0.29	0.30	−0.1	0.41	−0.05	0.60	−0.1	0.23	0.1	0.19	−0.04	0.68	0.2	0.33	−0.08	0.49	−0.1	0.33	0.1	0.31
Sleep tooth grinding	0.2	0.03	0.06	0.49	0.1	0.13	0.1	0.17	0.06	0.46	0.06	0.52	0.3	0.008	0.05	0.59	0.1	0.18	0.1	0.23
Fruits and vegetables	0.3	0.002	0.3	0.002	−0.02	0.87	−0.1	0.28	0.09	0.34	−0.05	0.60	0.2	0.09	0.8	0.02	−0.18	0.55	0.1	0.30
Breads and cereals	0.07	0.49	0.2	0.14	0.07	0.12	0.08	0.45	0.1	0.31	0.08	0.38	0.1	0.47	0.04	0.71	0.1	0.44	0.1	0.31
Milk products	0.2	0.06	0.01	0.90	0.04	0.67	0.02	0.88	0.05	0.62	−0.08	0.39	−0.03	0.82	−0.1	0.69	0.1	0.68	−0.3	0.03
Fried products	0.2	0.02	0.02	0.25	−0.003	0.97	−0.1	0.27	0.03	0.76	0.06	0.53	0.2	0.23	0.05	0.65	−0.1	0.57	0.1	0.48
Sweet and chocolates	−0.2	0.14	−0.06	0.55	−0.07	0.44	−0.1	0.18	−0.2	0.03	0.09	0.38	0.2	0.07	0.3	0.007	0.1	0.57	0.1	0.51
Sodas	−0.5	0.64	0.06	0.58	0.02	0.77	0.04	0.72	0.03	0.78	0.1	0.57	−0.2	0.09	−0.3	0.01	−0.2	0.25	−0.1	0.44
Alcohol	0.1	0.15	0.15	0.14	−0.02	0.81	0.01	0.96	0.01	0.93	0.02	0.81	0.2	0.15	0.2	0.04	0.2	0.23	0.2	0.04

**Table 3:** Correlation of age, gender, sleep tooth grinding and dietary-habits with non-occlusal/non-incisal tooth wear in permanent and deciduous teeth. Spearman correlation analysis (Rho Correlation Coefficients and p-values)

	Non-Occlusal/Non-Incisal tooth wear															
Variables	26		46		11		31		54		63		85		83	
	Rho	*p* value	Rho	*p* value	Rho	*p* value	Rho	*p* value	Rho	*p* value	Rho	*p* value	Rho	*p* value	Rho	*p* value
Age	0.2	0.05	0.3	0.002	0.07	0.42	−0.05	0.63	0.4	0.001	0.4	0.0003	0.4	0.002	0.4	0.002
Gender	−0.01	0.90	−0.2	0.06	0.006	0.94	0.03	0.75	0.08	0.49	−0.05	0.67	−0.09	0.43	0.1	0.60
Sleep tooth grinding	0.1	0.23	0.2	0.035	−0.02	0.78	0.1	0.27	−0.06	0.48	−0.03	0.75	0.14	0.22	0.3	0.007
Fruits and vegetables	0.7	0.02	0.04	0.70	−0.005	0.95	0.06	0.55	0.005	0.97	−0.1	0.61	−0.02	0.86	−0.1	0.39
Breads and cereals	0.02	0.82	−0.05	0.65	−0.02	0.80	−0.005	0.95	−0.1	0.38	−0.2	0.16	−0.1	0.38	−0.1	0.29
Milk products	−0.1	0.59	−0.06	0.56	0.06	0.52	0.02	0.84	−0.3	0.32	−0.2	0.08	−0.3	0.01	−0.2	0.18
Fried products	−0.03	0.76	0.02	0.82	−0.1	0.45	−0.1	0.29	−0.1	0.32	−0.2	0.12	−0.1	0.42	−0.2	0.08
Sweet and chocolates	0.002	0.97	−0.1	0.41	−0.3	0.005	−0.1	0.26	0.2	0.17	0.2	0.195	0.1	0.28	0.05	0.71
Sodas	−0.04	0.70	−0.1	0.50	0.02	0.81	0.7	0.02	−0.2	0.19	−0.2	0.20	−0.1	0.31	−0.2	0.27
Alcohol	−0.1	0.58	−0.10	0.32	−0.03	0.72	0.02	0.82	−0.002	0.84	−0.06	0.63	−0.06	0.62	−0.1	0.35

### Correlation between parental-reported STG and tooth wear

For parental-reported STG, a significant positive correlation was found with occlusal wear of teeth 16 (Rho = 0.20) and 64 (Rho = 0.29) (Table 2) and with non-occlusal/non-incisal wear of teeth 46 (Rho = 0.22) and 83 (Rho = 0.35) (Table 3). No statistically significant correlations were found for the other teeth.

### Correlation between dietary habits and occlusal/incisal and non-occlusal/non-incisal tooth wear

Findings showed that the occlusal/incisal wear of 16, 36, and 53 (Table 2), and the non-occlusal wear of 26 (Table 3) increase with the consumption of fruits and vegetables (Rho = 0.3 for occlusal/incisal wear of 16, 36 and non-occlusal wear of tooth 36, and Rho = 0.8 for incisal wear of tooth 53). Positive correlations were also found for sweets and chocolates with incisal wear of 53 (Rho = 0.3) (Table 2) and for sodas with non-incisal wear of 31 (Rho = 0.7) (Table 3).

Breads and cereals were not correlated with any of the tooth wear findings. As for milk products, the higher their consumption, the lower the occlusal wear in 75 (Rho = −0.3) and non-occlusal wear of 85 (Rho = −0.3). A similar finding was detected for sweets and chocolates, and sodas, which showed significant negative correlation with incisal wear of 41 (Rho = −0.2) and incisal of 53 (Rho = −0.3), respectively (Table 2).

Alcohol consumption was significantly correlated with incisal wear of 53 (Rho = 0.2) and occlusal wear of 75 (Rho = 0.2).

### Multiple-variable regression analysis

Ordinal multiple-variable regression analysis was not performed for teeth 12, 32, and 73, due to lack of variables with *p* < 0.1 in the single variable correlation analysis. Final ordinal multiple-variable models with some significant variables were obtained for occlusal/incisal tooth wear of 16, 36, 64, 53 and 75 and non-occlusal/non-incisal wear of 11, 54, 63, 85 and 83 (Table 4). None of the independent variables included for the models of teeth 21, 41, 26, 46, achieved significance values below 0.05; thus, final ordinal multiple-variable models were not possible to build.

**Table 4:** Association between dietary items and sleep tooth grinding with severity of tooth wear. Ordinal Multiple-variable logistic regression analysis

Dependent variables of the models (Tooth wear)	Independent Variables Included in the Model	OR	*p* value	CI	Influence of the Model on Tooth Wear	Ordinal OR	*p* value	CI	R^2^
Occlusal 16	Fruits and vegetables	1.67	0.003	1.18 - 2.35	1 vs 0^a^	2.96	NS	0.91 – 5.01	0.093
	Sweets and chocolates	0.73	0.04	0.54 – 0.99	2 vs 0^a^	6.54	S	3.67 - 9.41	
Occlusal 36	Fruits and vegetables	1.43	0.02	1.06 – 1.92	1 vs 0^a^	3.06	S	1.30-4.83	0.06
					2 vs 0^a^	6.52	S	3.85-9.17	
Occlusal 64	Age	1.48	0.02	1.05 – 2.07	1 vs 0^b^	5.87	S	2.00-9.73	0.08
	Fruits and vegetables	1.38	0.02	1.03-1.85	2 vs 0^b^	8.17	S	4.08-12.26	
					3 vs 0^b^	9.18	S	4.91-13.14	
Incisal 53	Age	1.54	0.006	1.13- 2.09	1 vs 0^b^	3.62	NS	0.77-6.46	0.04
					2 vs 0^b^	5.31	S	2.31- 8.31	
					3 vs 0^b^	7.39	S	4.12- 10.66	
Occlusal 75	Milk products	0.53	0.03	0.30-0.94	1 vs 0^a^	−1.92	NS	−5.46 - 1.61	0.094
	Alcohol	4.30	0.003	1.64- 11.26	2 vs 0^a^	6.54	NS	−2.67- 4.62	
Non-incisal 11	Sweets and chocolates	0.46	0.02	0.24-0.89	1 vs 0^a^	0.34	NS	−0.95-1.64	0.12
Non-occlusal 54	Age	2.21	0.002	1.32- 3.68	1 vs 0^a^	9.49	S	4.11-14.86	0.16
					2 vs 0^a^	11.12	S	5.49-16.75	
Non-incisal 63	Age	2.43	0.001	1.43- 4.12	1 vs 0^a^	10.22	S	4.70- 15.74	0.16
					2 vs 0^a^	11.63	S	5.90- 17.37	
No-occlusal 85	Age	2.29	0.003	1.33-3.97	1 vs 0^a^	9.87	S	4.13- 15.61	0.19
Non-incisal 83	Age	2.63	0.006	1.32- 5.23	1 vs 0^a^	11.69	S	4.27 – 19.11	0.21
					2 vs 0^a^	12.81	S	2.18 -20.44	

^a^0 = No wear; 1 = Wear within the enamel; 2 = Wear with dentine exposure

^b^0 = no wear; 1 = visible wear within the enamel; 2 = visible wear with dentin exposure and loss of clinical crown height of ≤1⁄3; 3 = loss of crown height > 1⁄3 but <2⁄3; and 4 = loss of crown height ≥ 2⁄3

Age was significantly correlated with wear of teeth 54, 63, 85 and 83 with OR > 2, indicating that an increase in age increases more than twice the risk of having non-occlusal/non-incisal wear in those deciduous teeth (Table 4).

Parental-reported STG was not included in the final ordinal multiple-variable regression models of any of the teeth, showing no influence of parental-reported STG on tooth wear of the mixed dentition in this study.

As for the dietary habits, tooth wear was significantly associated with the consumption of fruits and vegetables, sweets and chocolates, and milk products, even if OR values were lower than 2. Alcohol was found to be a risk factor for occlusal wear of tooth 75 (OR = 4.30) (Table 4).

With minor exceptions, the significant variables included in the final ordinal multiple-variable regression models increased progressively the severity of tooth wear (Ordinal OR > 2). The percentages of explained variance (Pseudo R^2^) were between 4% and 19%.

## Discussion

Tooth wear is an important outcome measure in medical and dental literature, as it may be a sign of some health conditions and/or a consequence of certain behaviors. The etiology of tooth wear involves several potential factors, among which sleep tooth grinding and dietary habits. Unfortunately, the reports on the effects of parental-report STG and dietary habits on the occurrence and severity of tooth wear are inconsistent, especially as far as the literature on children is concerned [4, 6, 13, 21].

This investigation assessed the relationship of parental-report STG and dietary habits with tooth wear in children with a mixed dentition. Efforts were made to increase the internal validity of the results, through the use of validated instruments to assess tooth wear and dietary habits as well as the adoption of parental-report STG as an approximation to “possible” sleep bruxism [22]. Other possible causes of tooth wear (e.g., neurologic or psychiatric disorders, and/or GERD) were excluded.

Overall, the findings suggest that: 1) Parental-report STG is not associated with tooth wear in the mixed dentition; 2) the consumption of fruits and vegetables, sweets and chocolates, milk products, and alcohol were found to be correlated with some tooth wear patterns, but the correlation values were weak; and 3) despite the observation that there is an association between dietary habits and the increase-to-increase severity of both occlusal/incisal and non-occlusal/non-incisal tooth wear in some teeth, the percentage of the explained variance for tooth wear with ordinal multiple-variable models is low.

Findings of this investigation are not in agreement with those of previous studies showing a positive association of parental-report STG and tooth wear in the mixed and permanent dentitions [6, 13, 21]. Indeed, despite the existence of a few significant correlations with parental-report STG (i.e., occlusal wear of tooth 16 and non-incisal wear of tooth 83), the correlation coefficients were very low. Moreover, no association with parental-report STG was found in any of the multiple-variable models. Regarding the primary dentition, our findings are in line with reports on samples of children, indicating no differences in tooth wear between individuals with and without “possible” sleep bruxism [23]. The findings of this investigation may have important implications in the light of common claims that tooth wear is a sign of “probable” sleep bruxism [6, 22].

Regarding the dietary habits, previous investigations assessing the risk factors for tooth wear in the primary, mixed, and permanent dentitions with questionnaires filled out by the parents, found an association with the consumption of soft drinks, non-natural juices, citrus flavored sweets/gums, and citrus fruits [4, 23]. Our results show correlations between wear of permanent molars and deciduous canines on the one hand and the consumption of fruits and vegetables, sodas, and sweets and chocolates on the other, but strength of the correlations is weak. Interestingly, when included in the ordinal multiple-variable models, it seems that some of those correlations are strong enough for an increase-to-increase relationship. For instance, an increase in the consumption of fruits and vegetables has a clinical influence on the increasing severity of occlusal wear of teeth 16, 36 and 64, whilst sweets and chocolates have the same effect on the severity of wear of 16, as shown in the multiple-variable analysis.

In addition to the above findings, an association of alcohol consumption with the wear of tooth 75 was shown. Even though the clinical relevance of this finding is yet to be explored and hard to explain, especially in the light of the single tooth being associated with -tooth wear, it serves as a silent witness of the consumption of alcohol at an early stage of life. The age at which alcohol consumption starts has dramatically reduced in the last decade and, on average, children in Latin America start drinking alcohol at the age of 10 [24]. This finding is in line with a previous study, which reported a positive correlation between the consumption of alcoholic drinks and the increasing prevalence of tooth wear in adolescents, thus suggesting that the topic is worth of further exploration [9].

As for other factors, describing the influence of age on tooth wear goes beyond the scope of this investigation, but it is noteworthy that aging emerged as the main factor associated with tooth wear, especially as far as deciduous teeth are concerned [1]. This finding was previously reported for the mixed dentition [25], thus suggesting that age has to be taken into account as an important co-factor when designing future investigations on the etiology of tooth wear in the different dentitions.

This study has limitations, mainly represented by the cross-sectional design, which prevents from drawing conclusions about the longitudinal course of tooth wear. The main difficulty when performing studies on this issue is the multifaceted nature of tooth wear phenomena [7, 26]. Mixed dentition is a variable condition, since the teeth eruption time is different among individuals. This must be considered as a study limitation, since the time the teeth erupted and stood in the mouth cavity -exposed to attrition, abrasion and erosion – could be one of the variables that should be included in further studies. Additionally, STG was based on parental-report, not with polysomnography, which is the gold-standard for the assessment of SB, but that has high technical requirements and costs.

In addition, validated instruments for a standardized evaluation of tooth wear are scarce. Recently, the TWES presented the module “qualification” to differentiate erosion, attrition, and abrasion, and the assessment of its reliability for use in deciduous teeth as well as its adoption in longitudinal investigations on tooth wear progression may help getting deeper into the topic of tooth wear etiology.

Taken together, the findings of this investigation suggest that issues different to parental-report STG and dietary habits are involved in the etiology of tooth wear in the mixed dentition. The explained variance of the regression models was low, thus other factors are to be included within the framework of multifactorial etiology. On the one hand, sociodemographic and cultural influences on the dietary habits [27], and their effects on tooth wear should be evaluated. On the other hand, the influence of the different patterns of interarch relationships during the often occlusally unsteady mixed dentition and of the functional jaw movements during craniofacial growth has to be considered in future studies, as only scarce literature exists on this topic [28, 29].

As a final remark, it should be pointed out that, according to this investigation, the use of tooth wear as a pathognomonic symptom of sleep bruxism in the mixed dentition is not sustained. Assessing tooth wear within a multiple-variable framework, including other etiological factors not assessed in this investigation (e.g., features of dental occlusion, techniques for oral hygiene, GERD) would lead to the buildup of a more reliable construct for its etiology. Such framework should be supported by prospective longitudinal studies.

## Conclusion

Tooth wear is an important measure of several health conditions and/or certain behaviors. Its etiology is multifactorial and there is a strong need for researches on the associated factors within a multiple-variable framework. Findings of this investigation did not show a strong correlation of dietary habits and sleep tooth grinding with tooth wear in the mixed dentition of healthy children, even if dietary habits seem to have effects in terms of increase-to-increase severity. Further investigations are needed to explore the phenomenon of tooth wear in longitudinal studies by also including other potentially relevant factors.

## Abbreviations

C.R: Claudia Restrepo
CSHQ: Children’s Sleep Habits Questionnaire
GERD: reflux disease
HBSC-FFQ: Health Behaviour in School-Aged Children Food-Frequency Questionnaire
OR: Odds Ratio
STG: parental-report sleep tooth grinding
TWES: Tooth Wear Evaluation System
